# Evaluation of boding between mother and child and mothers' mental health of children with mental illness

**DOI:** 10.1590/S1679-45082013000100012

**Published:** 2013

**Authors:** Custódia Virgínia de Nóbrega Mäder, Vera Lúcia de Alencar Monteiro, Patricia Vieira Spada, Fernando José de Nóbrega

**Affiliations:** 1 Stimulation and Habilitation Department, Associação de Pais e Amigos dos Excepcionais, São Paulo, SP, Brazil; 2 Graduate Program in Nutrition Department, Universidade Federal de São Paulo, São Paulo, SP, Brazil; 3 Hospital Israelita Albert Einstein, São Paulo, SP, Brazil; Graduate Program in Nutrition, Universidade Federal de São Paulo, São Paulo, SP, Brazil; Universidade Federal de São Paulo, Universidade Federal de São Paulo, São Paulo, SP, Brazil

**Keywords:** Mother-child relations, Maternal welfare, Intellectual disability

## Abstract

**Objective:**

To verify and evaluate the mother-child bond and mental health of mothers of children with intellectual disabilities.

**Methods:**

A total of 74 mothers of children aged up to 7 years participated. Data collection was made through interviews. Evaluation tools were Mother-Child Bonding Evaluation Protocol and Self-Report Questionnaire. We used statistical analysis χ^2^ and Student-*t* tests. A 5%-level of rejection of the null hypothesis was set.

**Results:**

There were no significant results between the average maternal ages, between bond and schooling, nor time of marriage and social status. The percentage of low social condition mothers with weak bond were 38.7% and in high condition, 68.8%.

**Conclusion:**

The occurrence of weak bond is associated with the Self-Report Questionnaire and socio-cultural conditions. That is, mothers with alteration in the Self-Report Questionnaire are more likely to develop mental disorders, weak bond with their children, the same occurring with the mothers in the most privileged social conditions.

## RESUMO

**Objetivo:**

Conhecer e avaliar o vínculo mãe-filho e a saúde mental de mães de crianças com deficiência intelectual.

**Métodos:**

Participaram 74 mães de crianças de até 7 anos com deficiência intelectual. A coleta de dados foi realizada por meio de entrevistas. Os instrumentos utilizados foram o Protocolo de Avaliação do Vínculo Mãe-Filho e o *Self-Report Questionnaire.* Para estatística, foram utilizados os testes de χ^2^ e *t* de Student. Fixou-se em 5% o nível de rejeição da hipótese de nulidade.

**Resultados:**

Não houve diferença significativa entre as médias das idades maternas, entre vínculo e escolaridade e nem entre tempo de união e condição social. A porcentagem de mães com vínculo fraco na condição socioeconômica baixa foi de 38,7% e na condição socioeconômica alta de 68,8%.

**Conclusão:**

A ocorrência de fraco vínculo está associada ao *Self-Report Questionnaire* e às condições socioculturais. Ou seja, mães com alteração no *Self-Report Questionnaire* têm maior probabilidade de desenvolver distúrbios mentais e maior chance de apresentar fraco vínculo com o filho, o mesmo ocorrendo com as mães nas condições sociais mais privilegiadas.

## INTRODUCTION

The birth of the first child is one of the most challenging events in life. It constitutes an opportunity for personal growth and to reach a special maturity level that only such event could bring. A child is a gift that gives the chance for someone to start a family. The desire for a son firstly answers an intuitive and visceral reproductive need being as well as an expression of love and wish to make a family with someone you love. For this reason, the birth of a child is a gift for those wishing to make or enlarge the family, so this is one of the best opportunities for such an achievement. The wish to have a child constitutes an important stage in a couple's life. After having a baby a woman and a man leave their place as daughters and sons and a new stage start in their relationship with their own parents presenting the chance for personal enrichment. However, the project of having a child can suffer various interferences such as how he was conceived, in which context, if he was planned, long expected and wished. The quality of this origin will follow the child's psychological life and will become part of his/her history. Besides, in the couple's imaginary, a son or daughter is object of desire or separation especially because ambivalent feelings are part of human beings and are also present during gestation^(1,2)^.

However, when a child is not consciously desired, the parents' feelings towards the child can be contradictory and may represent a risk to the emotional bonding of the parents with their child as well as or of the child with his/her parents^(3)^.

Boding can be usually defined as everything that ties up or unifies. In this paper the term “bonding” will be used as a symbolic representation of the emotional relationship that unifies one person to another. According to Nóbrega^(4)^, mother-child bonding is exteriorized based on different interferences and social influences concerning positive or negative characteristics of mothers, fathers or couples, and even of the child herself (her temper or personal characteristics) so that, it may compromise or not the bonding between parents and child. So, positive actions headed to a good bonding between mother and child, will always be useful and contribute for a more solid and happy relationship^(3)^.

Winnicott^(5)^ considers that even when a child is born healthy he/she will depend on a facilitating environment provided firstly by the mother because of her sensibility or intuition in favor of the child's mental health. The quality of bonding with the child depends on this sensibility. This status according to the author is related to motherhood, however, to reach this status a mother good mental health is required^(6)^.

Therefore, it is necessary a reflection concerning what can occur with the bonding when the child is born with any deficiency and this fact could develop opposite feelings because of what was expected by the parents^(7)^.

According to Coriat and Jerusalinsky^(8)^, “when a deficient child is born, the difference between the expected child and that baby who was actually born significantly affects the maternal role”.

Considering the facts previously reported it is possible to detect psychoemotional implications that change the maternal (or her substitute) behavior that may compromise the bonding with a mentally deficient child^(9,10)^.

It is also important to highlight the possibility to detect maternal psychological disorders, such as depression, personality disturbances, among others^(11)^.

Considering all these aspects, to understand the dynamics of the mother-child relationship is fundamental. Mothers with emotional problems have difficulty to perceive and take care of their children in daily life, or even to perform activities as playing, more specifically, when or where to play, as well as what kind of toys to choose for playing activities. The mother is the one who stays almost all the time with the child and satisfies his/her physical or physiological needs. In general, the mother is also the one in charge to present the world, things and people to her child. Above all, mothers are those who teach their children to deal with feelings^(12–14)^.

## OBJECTIVE

To verify and evaluate boding between mother and child and mothers' mental health of children with mental illness as well as to evaluate their emotional status and identify risk factors for the occurrence of a weak mother-child bonding.

## METHODS

This study was carried out at Associação dos Pais e Amigos dos Excepcionais (APAE) in São Paulo, Brazil. The Ethical and Research Committee of Universidade Federal de São Paulo (UNIFESP) approved the study under n.1.875-08. All mothers agreed to participate and signed the consent form.

The study population comprised 74 mothers of children aged between 0 and 7 years assisted in the Stimulation and Habilitation department of APAE in São Paulo, during attending special days, so called “joint effort”, in order to complete remaining vacancies.

The process of entrance in the Stimulation and Habilitation service of APAE could be unleashed by medical referral or by the child's own family initiative after having confirmed the diagnosis of neurological illness in the maternity hospital.

Children suspected of genetic syndromes or deficiencies are admitted into the institution with the laboratorial tests that were requested by their physicians. If necessary, the child will undergo complementary exams in the diagnostic center of APAE in São Paulo when vacancies are available. Tests can be also performed at the Sistema Único de Saúde (SUS) or at private institutions.

Medicines, when required, are prescribed by a specialist (neuropediatrician, infant psychiatrist, cardiologist, among others) who follows the child's case.

After being submitted to screening at the ambulatory by the social assistant and psychologist, infants or children are referred to the Stimulation and Habilitation department to be assisted and followed-up.

In this study the level 1 denoted low social status and was composed of 58 mothers who did not have conditions to afford health insurance plans; basically their child care was performed at SUS. Level 2 included 16 mothers who had a health insurance plan or good financial conditions for paying their child's treatment.

The following instruments were used:

1. protocol for assessment of mother-child bonding^(4)^ (Appendix 1) upon admission. This is a validated instrument composed of 13 yes or no questions. Positive answers “yes” denoted the presence of an attribute or indicator of weak bonding. After summing all “yes” answers, a score varying of 1-13 was obtained. A positive classification for weak bonding occurred if positive answers were ≥5. The mother's history during the interview was used as an indicator of the quality of the mother-child bonding. For the mothers significant events were their infancy, adolescence, gestation, delivery, post partum period and current situation (professional, personal, marriage and family satisfaction).
2. application of a protocol for assessment of maternal mental health upon admission of mother and child, the Self-Report Questionnaire (SRQ)^(15)^ (Appendix 2). This questionnaire of self-information is a screening instrument for mental illness that identifies non-psychotic disorders in the community. The instrument has 20 questions with affirmative or negative answers and its purpose is to track the mother's emotional status in the last 15 days. Summing all “yes” answers a score varying from 1-20 is obtained. A positive classification ≤8 indicated that the mother needed more specific assistance by a professional from the Psychological area.

These instruments were applied by pedagogues and psychologists. Those professionals performing interviews were previously trained in order to use the same kind of approach.

### Statistical analysis

The sample was described using tables with descriptive statistical values for quantitative variables (age and SRQ) divided according to bonding category (good and weak) and total. For qualitative variables such as formal education, duration of marriage and social conditions tables with frequency distribution and percentages of these variables were used in each bonding category and total.

The SRQ was analyzed in two ways: as a quantitative variable, that is, considering the score obtained in the questionnaire and as a qualitative variable in categories that were ≤8 or >8.

To verify the presence of an association between age and SRQ with bonding, their means were compared in the two categories of bonding using the Student's *t* test. To evaluate the contribution of risk factors to the occurrence of a weak bonding, a model of logistic regression was adjusted^(16)^, considering the occurrence of a weak bonding as a variable response, and the variables in which p value <0.20 was obtained in the association tests with maternal bonding, as explaining variables. To the adjusting model we adopted the method of selection of variables, the forward stepwise.

## RESULTS

The study included 30 male children aged from 1 month to 4 years and 44 female children aged from 1 month to 5 years.

Table 1 shows descriptive statistical values concerning maternal age in years by bonding category.

**Table 1:** Descriptive statistics for maternal age by bonding category

Bonding	Maternal age (years)					
	n	Mean	Standard deviation	Minimal	Medium	Maximus
Good	40	32.7	7.2	17	34	46
Weak	34	30.6	9.0	18	30	48
Total	74	31.7	8.1	17	31	48

No significant differences were seen between maternal mean age in both groups defined by boding (p=0.273).

Descriptive statistics for SRQ scores are presented in table 2. Means and median were higher in the weak bonding group than in the strong bonding group.

**Table 2:** Descriptive statistics for Self-Report Questionnaire by bonding category

Bonding	SRQ					
	n	Mean	Standard deviation	Minimal	Medium	Maximum
Good	40	4.3	2.9	0	4	11
Weak	34	7.6	4.0	0	7.5	17
Total	74	5.8	3.8	0	5	17

SRQ: Self-Report Questionnaire.

There was a significant difference between SRQ mean in both bonding groups (p<0.001). For this reason, it was possible to assume the presence of an association between bonding and SRQ, in other words, mothers with weak bonding had higher possibility to develop mental illness than mothers with good bonding.

Considering SRQ categories, table 3 was designed to present distributions of SRQ frequencies and percentages in each category of bonding. We observed that the percentage of mothers with changed SRQ was higher in the group with weak bonding.

**Table 3:** Distributions of frequencies and percentages of the Self-Report Questionnaire in each category of bonding

Bonding	SRQ		
	Changed n (%)	Normal n (%)	Total n (%)
Good	4 (10.0)	36 (90.0)	40 (100.0)
Weak	14 (41.2)	20 (58.8)	34 (100.0)
Total	18 (24.3)	56 (75.7)	74 (100.0)

SRQ: Self-Report Questionnaire.

There was association between bonding and SRQ (p=0.002). The percentage of mothers with weak bonding in the category of changed SRQ was 77.8% (14/18) and, in the normal category, it was 35.7% (20/56).

Regarding formal education only 2 mothers (2.7%) were illiterate and 14 (18.9%) had a college degree. Illiterate mothers and those with elementary education were included in the same group; the same was done with mothers with high school and college degree. The majority of mothers in both bonding groups had completed high school or college: good (28/40) and weak (18/34). A higher percentage of mothers with better education was seen in the group with good bonding. However, we did not find association between bonding and formal education (p=0.132).

Concerning duration of marriage no association was verified between bonding and duration of marriage (p=0.595).

Table 4 shows that most mothers had low social status in both bonding categories. Interestingly, the percentage of mothers in this status was higher in the good bonding group.

**Table 4:** Distribution of frequencies and percentages of social status in each bonding category

Bonding	Social status		
	1 n (%)	2 n (%)	Total n (%)
Good	35 (87.5)	5 (12.5)	40 (100.0)
Weak	23 (67.6)	11 (32.4)	34 (100.0)
Total	58 (78.4)	16 (21.6)	74 (100.0)

An association between bonding and social status (p=0.039) was seen. The percentage of mothers with weak bonding in level 1 (low socioeconomic status/SUS) was 39.7% (23/58) and in level 2 (high socioeconomic status/health insurance) was 68.8% (11/16).

## DISCUSSION

The findings of this study suggest that mother from low social status show more receptiveness in mother-child relationship. Based on these findings it is possible to raise the hypothesis that mothers who had little knowledge or information concerning mental illness and future expectations, as well as the possible difficulties of acceptance of a mentally deficient child by the society, were feeling that the situation is part of a definitive destiny in which they can only be near their child. It is possible that they perceive with simplicity and resignation the reality imposed. For this reason, these perceptions may work as a protective factor for the bonding with their children.

However, it is known that the impact of having a mentally deficient child can difficult and even prevent maternal reactions that, natural and intuitively, supply their child's needs.

This study showed that the majority of mothers from higher social classes tended to show a weak bonding with their children. One hypothesis to explain this finding is the fact that they have more information about the deficiency and are more conscious that despite the positive familiar environment, institutional resources and special schools, the child will have social difficulties and problems in learning, among others. The course of life may justify the little energy to accept the condition, interfering in the quality of bonding with their child.

Results of this study revealed that families go through different phases in which they learn to deal with the child that presents some type of special needs. According to Casarin^(17)^, some families live an acute crisis period but after a while they may recover. Other families may have more difficulties and develop a “chronic sadness”. The situation turns to be more difficult because the child will require specific care and high availability of caregivers. It is important to mention that when only one person delivers care for the child, a changing in the relationship with other family members may cause imbalance in the relationships and affect negatively on the quality of bonding between mother and the mentally deficient child, generating a vicious cycle which perhaps explains the results found in this study.

Mothers from high social status showed low energy, initiative and emotional investment, as well as, sometimes, deficient care with themselves, and often, with the child. Feelings of less value possibly interfere on the quality of bonding with the child as well as on the maternal and family behavior concerning the child's development. It is important to highlight that for the infant the process of understanding the world will depend, among other aspects, on his/her ability and possibility to explore it.

The deficient infant or child exploratory behavior depends on an adequate stimulation. According to Bowlby^(9)^, when the infant responds less or seems apathetic there is more chance of negligence because the child compensates less the mother and her behavior can change by the child's lack of reaction and vice versa.

For this reason, we can assure that the potential of each child's development will be better or worse depending on the opportunities offered by the environment where he/she is included. Perhaps these hypotheses justify the results found in this study.

It was also found that mothers with weak bonding showed in average a higher possibility to develop mental disorders than those with good boding.

We should consider what was stated by Vygotsky^(18)^: “since birth children gradually acquire their own meanings within their family context as well as they express their degree of physical or psychological development. It is by means of these activities that children start to become part of intellectual, mental and emotional life of those surrounding them, specially the mother, who is the main person to deliver care for them”.

To the mother the child means the continuation of her existence and, even before birth, there is a predetermined place in her mind where a number of expectations are placed. When the health status and the child's appearance do not correspond to what was imagined and idealized by the mothers it is very likely that they suffer a psychic rupture especially because the bonding with the child may not have been strong enough. As the child is seen as a projection of his/her parents, when he/she is different from expectations it could represent in a narcissist way loss of a dream or hope^(9)^. According to Voivodic^(19)^ “after a period of time, the disorganized family finds releif in the intense stimulation activities, but many times these activities can take the place of an affective relationship and the mother's availability to perceive and interact with the child”.

Because of the mother lack of emotional investment in the child her emotional health can be compromised as well as her child's health which explains the results found.

The quality of emotional bonding is built along the relationship. It is possible that when the mothers experience rejection feelings they feel guilty and have difficulty in understanding that affection occurs along a companionship process, among other factors. These feelings may lead them to develop significant conflicts because of the impossibility to differentiate the nonacceptance of the deficiency from their own child. Rejection affects feelings concerning the deficiency and may be present along the vital cycle. But mothers who developed healthy psychological resources can elaborate their life's condition - her real child- and acquire a better emotional structure to deal with this diversity.

We believe that mothers more emotionally committed can enhance the already existing difficulties in the relationship with their child because, perhaps, they take back the pain when remembering the moment they became aware of their child's syndrome. In addition, when their psychological status suggests depression and/or an important psychological change it is necessary to provide specific follow-up to the mother or the caregiver because these disorders interfere negatively and have harmful effects on global development of the child. It is important to highlight that depressive mothers are more vulnerable to emotional disorders, especially because they feel as incompetent mothers and, as a result, also evaluate their child negatively^(18)^, a fact that can also justify the results of this study.

Emotionally fragile mothers have close links with the feelings experienced by the lost of an “ideal” child and that could be expressed as shock, sadness, rejection, guilty and anger^(20,21)^.

Brazelton^(22)^ stated that to accept a deficient child is difficult for parents. Besides, each person needs a time to elaborate these feelings and this process has to be considered^(19)^. It is not possible to expect resignation from parents just after the birth of a deficient child, moreover, one cannot count that they will be available to participate promptly of an educational process^(19,23)^.

As stated by Regen^(14)^: “firstly there is need to help parents to understand that their feelings are normal. It is natural to be disappointed, depressed, feel pain, unsureness and fear, besides the wish that everything disappears as if it were a nightmare”.

We must emphasize that parents along time develop their own “listening” to the advices received and become able to apply and multiply these advices at home, school, or social environment. In addition, parents should assign priorities to actions that include their child and family in the society.

As it is, the role of this study as well as of stimulation and habilitation programs aiming to assist mothers, children and families of deficient child should be rethought. Stimulation and habilitation programs should assist caregivers during the adaptation process of taking care of children who have especial needs and also lead them concerning these needs as well as providing teaching and psychological support to the mother-child bonding in order to contribute to the health and global development of the child.

This investigation may also contribute to guide professionals working with mothers who have a deficient child. These professionals should help mothers to feel stronger and more valorized and, as a result, decrease the probability of mental disorders, which affects directly the quality of boding with the child.

### Limitations of the study

In this study we found an association between weak bonding and SRQ, which denoted that women with weak bonding in average had higher possibility to develop mental disorders than mothers with good bonding. However, a few mothers did not belong to the diagnostic criteria of SRQ, as they could present symptoms commonly classified as common mental disorders (CMD) which have high prevalence among women. We did not find in the studied population symptoms such as somatic complaints, fatigue, insomnia or excessive sleepiness, irritability, agitation, memory and concentration problems, feelings of guilt or self-devaluation, which could interfere in the results. It is possible that mothers who presented a weak bonding in this study had CMD and not symptoms that the SRQ suggests. The possibility to find an association between both could be considered a limitation of this study, but it does not invalidate the contributions provided in the study.

## CONCLUSION

This study enabled to evaluate and recognize weak or good mother-child boding according to emotional status of mothers assisted in the Stimulation and Habilitation department of APAE in São Paulo. In addition, it was possible to know some risk factors for the occurrence of weak mother-child bonding.

The weak bonding was associated with SRQ and to social status. Mothers with SRQ had more chance to present a weak bonding, the same was observed in mothers in high social status. There was no association with maternal age, formal education or duration of marriage.
